# Child socioemotional behavior and adult temperament as predictors of physical activity and sedentary behavior in late adulthood

**DOI:** 10.1186/s12889-023-16110-y

**Published:** 2023-06-19

**Authors:** Johanna Ahola, Katja Kokko, Lea Pulkkinen, Tiia Kekäläinen

**Affiliations:** 1 Gerontology Research Center, Faculty of Sport and Health Sciences, University of Jyväskylä, P.O. Box 35, Jyväskylä, FI-40014 Finland; 2 Department of Psychology, University of Jyväskylä, Jyväskylä, Finland

**Keywords:** Personality, Accelerometer, Longitudinal study, Life-span development

## Abstract

**Background:**

Most studies investigating the association of temperament with physical activity and sedentary behavior have examined children or adolescents, employed cross-sectional or longitudinal designs that do not extend from childhood into adulthood, and utilized self- or parent-reported data on physical activity and sedentary behavior. This longitudinal study investigated whether socioemotional behavior in childhood and temperament in middle adulthood predict accelerometer-measured physical activity and sedentary behavior in late adulthood.

**Methods:**

This study was based on the Jyväskylä Longitudinal Study of Personality and Social Development (JYLS). Socioemotional behavior (behavioral activity, well-controlled behavior, negative emotionality) was assessed at age 8 based on teacher ratings, whereas temperament (surgency, effortful control, negative affectivity, orienting sensitivity) was assessed at age 42 based on self-rating. Moderate-to-vigorous physical activity and sedentary behavior were assessed at age 61 using an accelerometer. Data (N = 142) were analyzed using linear regression analysis.

**Results:**

In women, behavioral activity at age 8 predicted higher levels of daily sedentary behavior at age 61. The association did not remain statistically significant after controlling for participant’s occupational status. In addition, women’s negative affectivity at age 42 predicted lower daily moderate-to-vigorous physical activity at age 61, particularly during leisure time. No statistically significant results were observed in men.

**Conclusions:**

Although few weak associations of socioemotional behavior and temperament with physical activity and sedentary behavior were detected in women, they were observed over several decades, and thus, deserve attention in future studies. In addition to other factors contributing to physical activity and sedentary behavior, health professionals may be sensitive to individual characteristics, such as a tendency to experience more negative emotions, when doing health counseling or planning for health-promoting interventions targeting physical activity and sedentary behavior.

**Supplementary Information:**

The online version contains supplementary material available at 10.1186/s12889-023-16110-y.

## Introduction

Individuals leading a more physically active and less sedentary lifestyle have a lower risk of developing several non-communicable diseases (e.g., coronary heart disease and type 2 diabetes) and facing premature death [1, 2]. However, globally more than one in four adults do not engage in the World Health Organization’s recommended amount of physical activity (PA), namely, at least 150 min of moderate PA, 75 min of vigorous PA, or a combination of these two intensities per week [3], which has remarkable economic consequences [4]. Moreover, adults in high-income countries spend the major part of their waking time being sedentary [5], which further increases the economic burden [6].

PA involves any bodily movements produced by skeletal muscles, causing the energy expenditure to exceed the basal metabolic rate [7]. By contrast, sedentary behavior (SB) is any waking behavior that is performed in a sitting, reclining, or lying posture that requires low energy (≤ 1.5 metabolic equivalents) [8]. The behaviors are interrelated within a 24-hour activity cycle together with sleep, meaning that an increase in one activity results in a decrease in another [9]. However, PA and SB deserve to be investigated separately for two focal reasons. First, they are independent factors associated with several health-related outcomes, including all-cause mortality [1, 2], although PA may reduce the health risks caused by SB [9]. Second, SB may be commonly accumulated a lot even when the recommended amount of PA is met; thus, SB does not equate to physical inactivity [1].

In the 21st century, worrying trends in the levels of PA and SB have been observed, with the prevalence of physical inactivity and SB increasing in high-income countries [3, 10]. Nowadays, SB can be difficult to avoid owing to several perpetuating social and environmental factors, such as sedentary jobs [11] and less physically demanding domestic tasks [12] that have become more common. PA and SB can indeed occur in the leisure (e.g., exercise), occupational (e.g., manual labor tasks), transportation (e.g., active commuting), and domestic (e.g., housework) domains [13]. Although current recommendations do not take these domains into account, an increasing amount of literature suggests that while leisure-time PA is beneficial to health [14], occupational PA is related to adverse health outcomes (e.g., an increased risk of early mortality) [15]. Hence, information on the domain-specific correlates of PA and SB are needed to develop targeted health-promoting interventions.

Personality characteristics (e.g., socioemotional behavior and temperament) may explain inter-individual differences in the levels of PA and SB even after a long time, as they have been reported to predict multiple behaviors related to health [16] and work life [17] after decades. Socioemotional behavior and temperament describe the basic dispositions regarding one’s feelings, reactions, and efforts to regulate arising reactions and the concepts share the same understanding that individual differences arise from the interaction between reactivity and self-regulation [18, 19]. Specifically, Pulkkinen (originally Pitkänen [20]) defined *socioemotional behavior* as the expression and regulation of one’s emotions in social relationships and characterized it by three higher-order dimensions: behavioral activity, well-controlled behavior, and negative emotionality [18, 21]. Similarly, Rothbart et al. [19, p. 123] defined *temperament* a few decades later as the relatively persistent “individual differences in reactivity and self-regulation” and conceptualized it as having three higher-order dimensions in childhood: surgency, effortful control, and negative affectivity.

These three dimensions are conceptual counterparts of each other [18]. Behavioral activity refers to one’s tendency to be actively in contact with others [18], while surgency refers to one’s tendency to show high activity, prefer high-intensity activities, and not feel uneasy in new social situations [19]. Well-controlled behavior refers to one’s tendency to act constructively and compliantly when facing a conflict [18], while effortful control refers to one’s tendency to regulate attention and behavior and prefer low-intensity activities [19]. Negative emotionality refers to one’s tendency to display both aggressive and anxious behaviors [18], while negative affectivity refers to one’s tendency to frequently experience feelings of sadness, discomfort, anger, and frustration [19]. Temperament in adulthood also includes the dimensions of orienting sensitivity and affiliativeness [22]. Orienting sensitivity refers to one’s tendency to sense cues from the external and internal environment, while affiliativeness refers to one’s tendency to respond empathetically to others’ feelings [22].

In previous studies, the links to PA and SB were examined using the concept of temperament. Studies investigating the associations of temperament with PA and SB used various measures and assigned different names to the temperament dimensions despite their similarities with existing ones [19], resulting in the difficulty of drawing definitive interpretations of the literature. Recent evidence, however, suggests that child surgency and related temperamental activity are associated with greater PA [23–26] and lower levels of SB in childhood [23, 27] as well as predict greater PA in adolescent boys [28]. However, child temperamental activity predicted lower PA and higher levels of SB in men within a follow-up period of over 20 years [26]. Negative affectivity is, in turn, linked to lower PA in boys [24], and a similar association was observed in men in the Jyväskylä Longitudinal Study of Personality and Social Development (JYLS) [29]. In a follow-up study related to Sharp et al. [24], this dimension predicted lower PA in girls and greater PA in boys [30]. Studies on child effortful control and related well-controlled behavior reported the most inconsistent results, wherein these dimensions were associated with lower PA and higher levels of SB in childhood [23] but predicted greater PA in women in the JYLS [16]. Orienting sensitivity is, in turn, linked to greater PA in adulthood [29].

Despite some conflicting findings, temperament has been suggested to be a relevant factor for PA and SB at different ages, and it may have predictive value for these behaviors over decades. However, the majority of the previous studies examined children [23–25, 27, 30] or adolescents [25, 28], employed cross-sectional [23, 24, 27] or longitudinal designs that do not extend from childhood into adulthood [28–30], and utilized self- or parent-reported data on PA and SB [16, 24, 26, 28–30]. The domains of PA and SB also varied across studies that focused either on the investigation of leisure time by using questionnaires [16, 24, 26, 28–30] or the assessment of non-domain-specific activities by using accelerometers [23, 25, 27]. Compared to questionnaires that tend to underestimate SB [5], accelerometers provide detailed information of intensity, frequency and duration of also habitual and incidental physical movements, which may be difficult to memorize [13]. None of the previous studies on the links between temperament and PA or SB used accelerometers to assess the PA and SB of adults and, simultaneously, several domains of PA and SB (e.g., leisure and occupational domains).

This study aimed to fill the current research gaps, with the major objective of investigating whether socioemotional behavior in childhood (age 8) and temperament in middle adulthood (age 42) predict PA and SB in late adulthood (age 61). In particular, this study aimed to assess the associations of multiple dimensions of child socioemotional behavior and adult temperament with accelerometer-measured moderate-to-vigorous physical activity (MVPA) and SB. In addition to whole-day MVPA and SB, leisure and occupational domains were investigated. Having data on inter-individual differences from two phases of life enabled the investigation on whether MVPA and SB can already be predicted by personality characteristics in childhood or only in adulthood.

On the basis of previous findings [23–26, 28–30], in this study, it was hypothesized that behavioral activity, surgency, and orienting sensitivity are associated with greater MVPA, whereas negative emotionality and negative affectivity are associated with lower MVPA. It was also hypothesized that these links exist from child socioemotional behavior into MVPA and SB in late adulthood but may be stronger when analyzed within adulthood in a shorter time interval. The literature on the association of well-controlled behavior and effortful control with PA [16, 23] and of all dimensions with SB [23, 26, 27] remains inconsistent. Setting unambiguous hypotheses is difficult because of these inconsistent findings based on various measures and because none of the previous studies followed their participants for five decades. This study aimed to supplement the previous studies based on the JYLS [16, 29] by extending the follow-up period to late adulthood (8–50 years old vs. 8–61 years old) and adopting a new method to assess PA (self-reporting vs. accelerometer-based measurement).

## Methods

### Study design and participants

This study was based on the JYLS [21], particularly on its most recent data collection called TRAILS (Transitions at Age 60: Individuals Navigating Across the Lifespan) [31]. For the first data collection in 1968, the participants (initial N = 369, 53% males) were drawn from randomly selected second grade classes in schools located in the town center and suburban areas of Jyväskylä, Central Finland. The selection method enabled the gathering of a representative sample without initial attrition. All participants were native Finns, and nearly all (94%) were born in 1959 [21].

Data were collected in several major waves: at ages 8, 14, 27, 36, 42, 50, [21] and 61 years [31]. The current study used the longitudinal data collected in 1968, 2001, and 2020–2021 when the participants were aged 8, 42, and 61 years, respectively. The initial study plan to collect teacher ratings was approved by the local school authorities, and the adult participants agreed to participate each time by signing a written informed consent [21]. In 2001, the Ethical Committee of the Central Finland Health District approved the data collection (42/2000) [32], and in 2020–2021, the procedures were approved by the Ethical Committee of the University of Jyväskylä (12/13/2019) [31]. In the current study, data were obtained from teacher ratings, self-ratings, and accelerometer measurements. The accelerometer measurements were conducted amid the COVID-19 pandemic in 2020–2021 but not during the state of emergency in spring 2020. Finland had mild restrictions (e.g., no curfew), and possibilities for outdoor physical activities among 60-year-olds were favorable [33].

The study sample consisting of 42-year-olds represented the Finnish age cohort born in 1959 in terms of several demographic characteristics [32]. Those who remained in the study at age 61 were still representative of the same-age Finnish cohort [31]. The analytical sample in this study consisted of 142 participants who provided valid accelerometer data at age 61. Temperament data at age 42 were available for 131 of the 142 participants.

### Measures

*Child socioemotional behavior* was assessed at age 8 using the teacher ratings on 36 items, from which 27 items were used to measure three dimensions: *behavioral activity* (3 items; e.g., “Always busy and plays eagerly with other children during breaks and after school hours”), *well-controlled behavior* (constructiveness, 4 items; compliance, 3 items; emotional stability, 1 item; e.g., “Tries to act reasonably even in annoying situations”), and *negative emotionality* (aggressiveness, 8 items; anxiety, 3 items; low self-control, 5 items; e.g., “Teases smaller and weaker peers when angry at something”) [18]. The teachers were instructed to observe their pupils during breaks and rate their typical behavior on a four-point scale, with 0 representing “*does not apply at all to the pupil in question*” and 3 representing “*is very typical of the pupil in question*”. The girls were rated in relation to other girls, and the boys were rated in relation to other boys. The mean score for each dimension was computed, and a higher mean score indicated a stronger reflection of the socioemotional dimension in question. The factor analysis of the original sample indicated good internal consistency in all dimensions (α = 0.77–0.91) [18]. Teacher ratings were shown to be a valid method in the assessment of inter-individual differences in children at age 8 when peer nominations were used as criteria [21].

*Adult temperament* was assessed at age 42 using a self-reporting instrument. The short version of the Adult Temperament Questionnaire comprised 77 items assessing four dimensions: *surgency* (sociability, 5 items; high intensity pleasure, 7 items; positive affect, 5 items), *effortful control* (activation control, 7 items; attentional control, 5 items; inhibitory control, 7 items), *negative affectivity* (fear, 7 items; frustration, 6 items; sadness, 7 items; discomfort, 6 items), and *orienting sensitivity* (neutral perceptual sensitivity, 5 items; affective perceptual sensitivity, 5 items; associative sensitivity, 5 items) [22]. The items were answered on a seven-point Likert scale, with 1 representing “*extremely untrue of you*” and 7 representing “*extremely true of you*”. The mean score for each dimension was computed, and a higher mean score indicated a stronger reflection of the temperament dimension in question. The factor analysis based on a larger sample indicated good internal consistency in all dimensions (α = 0.79–0.83) [18, 29].

*MVPA* and *SB* were measured at age 61 using a triaxial accelerometer (UKK RM42) (UKK Terveyspalvelut Oy, Tampere, Finland). In this study, SB refers to time spent in sedentary behaviors but also to time spent in standing without ambulation, since the body posture was not measured with the current method [8]. Accelerometers were offered to those participants interested in health examination (N = 179), and data were collected between March 2020 and May 2021. The timing of the assessment period was flexible and targeted for a usual week of their life (e.g., postponed due to a holiday or sick leave). The participants were instructed to wear the device on a hip-worn elastic belt during waking hours for seven consecutive days, except when engaging in water-related activities. The participants used a diary to report their waking hours, sleeping time, times of starting and finishing work, non-step-based activities (e.g., cycling and swimming), periods when the accelerometer was removed for longer than 30 min and possible unusual events occurring during the week. The device and the diary were returned via mail.

The accelerometer sampling rate was 100 samples per second (Hz). The mean amplitude deviation (MAD) was calculated from the raw acceleration data [34], and a custom-written script on MATLAB (version R2016b, The MathWorks Inc., Natick MA, USA) was used to produce an average of 60 s MADs from non-overlapping epochs of 5 s. Intensity levels were defined as < 0.0167 g for SB and ≥ 0.091 g for MVPA [34, 35]. Non-wear time was defined as ≥ 120 min of continuous MAD values of < 0.02 g after the comparison of the analyzed wear times with diary-reported wear times on a sub-sample during the data collection (r = 0.83, N = 127).

Among the 149 participants who agreed to wear the accelerometer, 142 provided valid data for at least four valid days defined as a minimum of 10 h (600 min) a day [36]. Compliance to wearing the device was high, with 82% of the participants providing valid data for seven days. Working hours were based on diary reports, and leisure time was calculated by subtracting the working time from the whole-day wear time. The mean daily minutes of MVPA and SB for a whole day (N = 142), during leisure time (leisure-time MVPA / SB, N = 141), and when at work (occupational MVPA / SB, N = 99) were used as the main variables.

**Fig. 1:**
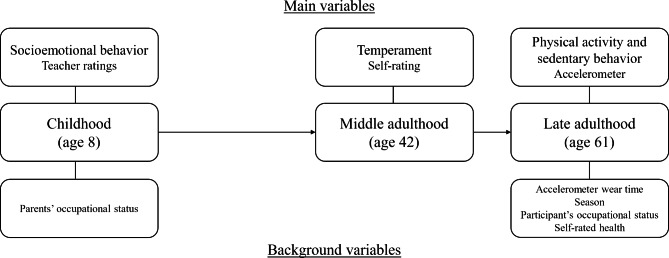
Summary of the variables and measures used in the longitudinal study

Accelerometer wear time, season, occupational status of parents and the participants, and self-rated health were used as background variables. The season of the accelerometer measurement period was dummy-coded to summer (June–August) and other months of the year. The occupation of the children’s parents (mainly based on the father’s occupation, with the exception of mothers who were the sole providers) or the adult participants was classified as either a blue-collar, a lower white-collar, or an upper white-collar job [16]. The occupations of the children’s parents at age 8 were reported by the children’s teachers, while the latest occupation of the participants at age 61 was self-reported. Occupation variables were dummy-coded by computing two variables (lower white-collar jobs vs. others; upper white-collar jobs vs. others). Self-rated health was assessed using a single question: “What has your state of health been during the past year?” [37]. Self-rating was based on a five-point scale, with 1 representing “*very good*” and 5 representing “*very poor*”. A reversed variable was computed. All variables and measures used in the current study are seen in Fig. 1.

### Statistical analyses

All statistical analyses were performed using the IBM SPSS Statistics version 26. Previous studies based on the JYLS [29] and other datasets [26] have reported gender-specific results regarding the associations of temperament with PA and SB. Thus, statistical analyses were performed separately for women and men.

The descriptive statistics are expressed as means and standard deviations for continuous variables and as percentages for categorical variables. In comparing the two genders in terms of the means of continuous variables, independent samples t-test was used, whereas differences in frequencies of categorical variables were tested with chi-square test. Pearson bivariate correlations were computed, and Fisher’s z-test was conducted to obtain the correlation differences between women and men.

Linear regression analysis was used as the main statistical analysis to examine the associations between the main variables. Owing to the positively skewed distributions, square root transformations of whole-day MVPA and leisure-time MVPA and a cube root transformation of occupational MVPA were used in the analyses. Regression models were adjusted for accelerometer wear time, season, occupational status, and self-rated health. The models incorporating child socioemotional dimensions were adjusted for the parents’ occupational status and additionally for the participant’s own occupational status, whereas the models incorporating adult temperament dimensions were adjusted for the participant’s occupational status. For sensitivity purposes, additional linear regression models were produced by excluding those participants whose accelerometer measurement overlapped with the declared COVID-19-related state of emergency in Finland (March 16, 2020–June 16, 2020) (N = 11).

The effect size of the standardized beta-coefficient was considered to be small when β = 0.10–0.29, medium when β = 0.30–0.49, and large when β ≥ 0.50 [38]. A p-value of < 0.05 indicated statistical significance. Additionally, analyses were corrected for multiple comparisons using the Benjamini*–*Hochberg method [39]. A false discovery rate of 0.10 was used to assess the significance of 20 gender comparisons in Table 1. Similarly for the regression analyses, the significance of 12 p-values of childhood analysis (2 outcomes and 3 predictors by gender) in Table 2, and 16 p-values of adulthood analysis (2 outcomes and 4 predictors by gender) in Table 3 were calculated separately for each model (Models 1, 2, and 3), including different sets of covariates.

## Results

The descriptive statistics for all participants and for only the women and men are presented in Table 1. As reported in previous JYLS publications that analyzed slightly different samples [18, 29], boys scored higher than girls for negative emotionality, while women scored higher than men for negative affectivity and orienting sensitivity. No gender-based differences were observed in terms of MVPA and SB. Among those who reported working hours (N = 99, 62% women), men spent more time at work than did women.

**Table 1:** Descriptive statistics

			All		Women		Men		Gender difference		
			N	Mean (SD)	N	Mean (SD)	N	Mean (SD)	t ^a^	df	p
Socioemotional behavior (0–3)											
	Behavioral activity		142	2.09 (0.73)	78	2.19 (0.71)	64	1.97 (0.73)	1.81	140	0.073
	Well-controlled behavior		142	1.50 (0.72)	78	1.58 (0.71)	64	1.40 (0.74)	1.50	140	0.135
	Negative emotionality		142	0.48 (0.44)	78	0.39 (0.38)	64	0.60 (0.48)	–2.81	118	0.006
Temperament (1–7)											
	Surgency		131	4.30 (0.71)	73	4.33 (0.64)	58	4.26 (0.78)	0.59	129	0.555
	Effortful control		131	4.87 (0.61)	73	4.89 (0.57)	58	4.85 (0.65)	0.36	129	0.722
	Negative affectivity		131	3.72 (0.69)	73	3.94 (0.62)	58	3.44 (0.69)	4.35	129	< 0.001
	Orienting sensitivity		131	4.76 (0.79)	73	4.95 (0.73)	58	4.52 (0.80)	3.19	129	0.002
Physical activity (min/day)											
	Whole-day MVPA		142	55.25 (31.37)	78	54.11 (31.49)	64	56.64 (31.42)	–0.48	140	0.634
	Leisure-time MVPA		141	44.39 (27.87)	78	43.78 (26.52)	63	45.14 (29.65)	–0.29	139	0.776
	Occupational MVPA		99	21.45 (18.81)	61	19.32 (19.03)	38	24.87 (18.17)	–1.44	97	0.154
Sedentary behavior (min/day)											
	Whole-day SB		142	514.32 (104.55)	78	527.65 (93.22)	64	498.09 (115.56)	1.69	140	0.094
	Leisure-time SB		141	373.59 (130.21)	78	366.01 (131.32)	63	382.97 (129.25)	–0.77	139	0.444
	Occupational SB		99	289.72 (125.33)	61	302.28 (121.97)	38	269.56 (129.62)	1.27	97	0.208
Wear time (h/day)											
	Whole-day wear time		142	14.69 (1.10)	78	14.70 (1.00)	64	14.67 (1.23)	0.13	140	0.898
	Leisure-time wear time		141	10.82 (2.82)	78	10.65 (2.74)	63	11.03 (2.91)	–0.78	139	0.435
	Occupational wear time		99	7.84 (1.91)	61	7.50 (1.84)	38	8.37 (1.93)	–2.26	97	0.026
Self-rated health (1–5)			142	3.92 (0.83)	78	3.96 (0.75)	64	3.87 (0.93)	–0.60	119	0.549
				**%**		**%**		**%**	**x** ^**2 b**^	**df**	**p**
Season			142		78		64		0.07	1	0.866
	Summer		67	47.2	36	46.2	31	48.4			
	Fall, winter or spring		75	52.8	42	53.8	33	51.6			
Parents’ occupational status			142		78		64		9.63	2	0.007
	Blue collar		99	69.7	59	75.6	40	62.5			
	Lower white-collar		33	23.2	11	14.1	22	34.4			
	Upper white-collar		10	7.0	8	10.3	2	3.1			
Occupational status			142		78		64		43.00	2	< 0.001
	Blue collar		33	23.2	4	5.1	29	45.3			
	Lower white-collar		64	45.1	52	66.7	12	18.8			
	Upper white-collar		45	31.7	22	28.2	23	35.9			

*Note.* MVPA = moderate-to-vigorous physical activity, SB = sedentary behavior, N = number, SD = standard deviation, t = t-value, df = degrees of freedom, p = p-value, x^2^ = chi-square value. All statistically significant p-values (p < 0.05) remained statistically significant after the Benjamini–Hochberg correction.

^a^ independent samples t-test

^b^ chi-square test

The Pearson bivariate correlations for the main and background variables are presented in the Additional file 1 (Table S1). Based on the correlation matrix, in women, higher scores for negative affectivity correlated with lower whole-day MVPA (r = − 0.28) and leisure-time MVPA (r = − 0.27), while higher scores for surgency correlated with greater leisure-time MVPA (r = 0.28). In men, higher scores for effortful control and lower scores for negative affectivity correlated with higher levels of occupational SB (r = 0.38, r = − 0.42).

Linear regression models were used to examine the associations of personality characteristics with whole-day MVPA and SB. In the analysis of child socioemotional dimensions, after controlling for season, parents’ occupational status and self-rated health, higher scores for behavioral activity predicted higher levels of daily SB in women (Table 2). The association was small (β = 0.24, p = 0.035) and did not remain statistically significant either after the Benjamini–Hochberg correction or the additional adjustment with the participant’s occupational status. In the analysis of adult temperament dimensions, higher scores for negative affectivity predicted lower daily MVPA in women (Table 3). The association was small (β = −0.27, p = 0.028) and remained statistically significant after controlling for season, participant’s occupational status and self-rated health. The associations were not statistically significant after the Benjamini–Hochberg correction. No other statistically significant associations were found.

**Table 2:** Linear regressions of child socioemotional dimensions predicting whole-day MVPA and SB.

		Whole-day MVPA									Whole-day SB									
		Model 1 ^a^			Model 2 ^b^			Model 3 ^c^			Model 1 ^a^			Model 2 ^b^			Model 3 ^c^			
		β	p	R^2^_adj__._	β	p	R^2^_adj__._	β	p	R^2^_adj__._	β	p	R^2^_adj__._	β	p	R^2^_adj__._	β	p	R^2^_adj__._	
Women				0.08			0.14			0.11			0.08			0.15			0.20	
	Behavioral activity	0.04	0.714		–0.02	0.842		–0.02	0.874		0.19	0.100		**0.24**	**0.035**		0.18	0.102		
	Well-controlled behavior	0.03	0.765		0.10	0.396		0.10	0.407		0.06	0.618		0.05	0.656		0.06	0.624		
	Negative emotionality	–0.04	0.752		0.02	0.834		0.02	0.865		–0.01	0.956		–0.05	0.636		–0.06	0.607		
Men				0.03			0.15			0.22			0.10			0.13			0.10	
	Behavioral activity	0.01	0.930		0.03	0.829		–0.04	0.774		0.09	0.501		0.07	0.577		0.07	0.585		
	Well-controlled behavior	–0.15	0.307		–0.21	0.134		–0.22	0.116		–0.17	0.223		–0.12	0.405		–0.11	0.450		
	Negative emotionality	–0.09	0.550		–0.02	0.911		–0.06	0.657		–0.04	0.781		–0.05	0.727		–0.05	0.765		

*Note.* Socioemotional dimensions analyzed in same regression models. Women N = 78, men N = 64. Square root transformation of whole-day MVPA was used. MVPA = moderate-to-vigorous physical activity, SB = sedentary behavior, β = standardized beta-coefficients, p = p-value, R^2^_adj__._ = adjusted coefficient of determination for the model. Statistically significant results bolded. None of the standardized beta-coefficients remained statistically significant after the Benjamini–Hochberg correction.

^a^ Model 1: Adjusted for accelerometer wear time

^b^ Model 2: Adjusted for accelerometer wear time, season, parents’ occupational status, and self-rated health

^c^ Model 3: Adjusted for accelerometer wear time, season, parents’ occupational status, self-rated health, and participant’s occupational status

**Table 3:** Linear regressions of adult temperament dimensions predicting whole-day MVPA and SB.

		Whole-day MVPA						Whole-day SB						
		Model 1 ^a^			Model 2 ^b^			Model 1 ^a^			Model 2 ^b^			
		β	p	R^2^_adj__._	β	p	R^2^_adj__._	β	p	R^2^_adj__._	β	p	R^2^_adj__._	
Women				0.19			0.21			0.04			0.12	
	Surgency	0.11	0.402		0.15	0.276		–0.04	0.785		–0.06	0.702		
	Effortful control	–0.05	0.686		–0.06	0.610		0.16	0.190		0.09	0.451		
	Negative affectivity	**–0.27**	**0.028**		**–0.27**	**0.034**		0.12	0.379		0.14	0.297		
	Orienting sensitivity	0.12	0.358		0.05	0.709		0.01	0.921		0.01	0.954		
Men				–0.01			0.19			–0.04			0.14	
	Surgency	–0.01	0.979		–0.13	0.484		0.06	0.775		0.04	0.834		
	Effortful control	–0.10	0.617		–0.19	0.310		0.03	0.895		–0.03	0.872		
	Negative affectivity	–0.07	0.731		–0.09	0.659		–0.03	0.901		–0.16	0.440		
	Orienting sensitivity	0.11	0.564		0.07	0.696		–0.02	0.920		0.10	0.603		

*Note*. Temperament dimensions analyzed in same regression models. Women N = 73, men N = 58. Square root transformation of whole-day MVPA was used. MVPA = moderate-to-vigorous physical activity, SB = sedentary behavior, β = standardized beta-coefficients, p = p-value, R^2^_adj__._ = adjusted coefficient of determination for the model. Statistically significant results bolded. None of the standardized beta-coefficients remained statistically significant after the Benjamini–Hochberg correction.

^a^ Model 1: Adjusted for accelerometer wear time

^b^ Model 2: Adjusted for accelerometer wear time, season, participant’s occupational status, and self-rated health

The associations of personality characteristics with leisure-time and occupational MVPA and SB were further analyzed (Supplementary Material, Tables S2–S5). Although behavioral activity was linked to higher levels of daily SB in women (Table 2, Model 2), it was not statistically significantly associated with either leisure-time (Table S2) or occupational SB (Table S3). Domain-specific analyses, however, revealed that the inverse association of the women’s negative affectivity with MVPA was apparent during leisure time (β = −0.27, p = 0.040) (Table S4). The small association remained statistically significant after controlling for season, participant’s occupational status and self-rated health (β = −0.25, p = 0.045) but not after the Benjamini–Hochberg correction. No new associations were detected in the domain-specific analyses. Sensitivity analyses indicated that exclusion of the participants whose accelerometer measurement period overlapped with the declared COVID-19-related state of emergency in Finland did not change the results.

## Discussion

This longitudinal study examined whether socioemotional behavior in childhood and temperament in middle adulthood predict accelerometer-measured PA and SB in late adulthood. Overall, behavioral activity at age 8 predicted higher levels of daily SB at age 61 in women. However, the association did not remain statistically significant after controlling for participant’s occupational status. Negative affectivity at age 42 predicted, in turn, lower daily MVPA at age 61 in women. This association was observed particularly during their leisure time. In men, personality characteristics were not associated with MVPA and SB.

Slightly unexpectedly, girls who were not withdrawn or timid and were busy and played eagerly with other children spent more time sedentary in late adulthood compared with their socially more passive peers. Compared to previous studies from Finland, this result is in conflict with those ones reporting a negative association of surgency with SB among preschool-aged children [23, 27] but in line with the one reporting child temperamental activity as a positive predictor of men’s TV viewing assessed over two decades later [26]. In that study, unadjusted models were also statistically significant for women [26]. Even though the effect sizes of the current findings based on standardized betas were small, they are in line with previous studies assessing longitudinal associations between child personality characteristics and adult health behaviors [16, 26, 40]. In the current study, controlling for parents’ occupational status strengthened the association of behavioral activity with SB. However, the statistically significant association was attenuated following additional adjustment for the participant’s own occupational status. In light of the finding, it seems that socially more active girls have higher occupational statuses in adulthood which are further associated with more SB accumulated during the day. In the previous study based on JYLS, the frequent contacts of girls with other children, namely social activity, was linked to high career orientation, including for example occupational status and education, in adulthood [41]. Higher occupational class has also been linked to higher levels of accelerometer-measured SB [42], providing additional support for the possible developmental path. Furthermore, the role of occupational status on the longitudinal association of behavioral activity with SB explains the disparity with the results of cross-sectional studies conducted in early childhood [23, 27] where social characteristics may also be expressed as active playing with peers.

Consistent with the hypothesis, women who experienced more negative emotions (e.g., frustration) in middle adulthood engaged less in MVPA in late adulthood, particularly during their leisure time, compared with women who experienced less of such emotions. The direction of the observed association is in line with previous findings in childhood [24, 30] and adulthood [29] and is further supported by studies reporting a consistent inverse association of equivalent effect size between conceptually similar personality trait neuroticism and MVPA [43, 44]. As discussed in the literature regarding the associations of personality traits with PA, the findings could be explained by the tendency of women who display high negative affectivity to experience displeasing emotions, such as discomfort, which might, in turn, impact how they enjoy less MVPA or how they prefer other types of lower-stimulus activities [45]. The fear of embarrassment may also serve as a barrier to engaging in intensive PA [46], while negative emotions may be associated with less autonomous motivation toward PA which, in turn, leads to less incidental PA [47]. The latter may also explain why the current findings were observed in women, although Karvonen et al. [29] found a negative relationship between negative affectivity and self-reported overall and vigorous PA in men participating in the same longitudinal study. In addition to the differences in sample sizes and lengths of follow-up, personality characteristics may be differently related to habitual and incidental PA captured by accelerometers compared to more deliberate PA captured by self-reports [43].

Although a follow-up period of several decades increases the remarkability of the current findings, it might also be a focal reason why only a few associations were detected in women and none in men. In men, there might be other factors (e.g., social support) associated with PA and SB that are more important, given that they are complex behaviors related to multiple individual, social, and environmental factors [48, 49]. Compared to the more deliberate PA, habitual and incidental physical movements may also be even more challenging to predict. Overall, only a maximum of one fifth of the variance for daily MVPA and SB was explained by the personality characteristics and covariates.

The strengths of this study include a uniquely long-term longitudinal design, along with the assessment of personality characteristics both in childhood and adulthood and the use of accelerometer-based measurement of PA and SB, with a high compliance rate in the wearing of the device. Accelerometers can monitor temporally accurate information on the intensity, frequency and duration of physical movements [13]. A limited number of participants who provided detailed diary reports also enabled the extraction of leisure and occupational times from the data.

Several limitations must also be acknowledged. First, there are risks for type I and II errors due to a relatively large number of tests and due to a relatively small sample size, respectively. However, in JYLS, the original sample had no initial attrition and relatively small attrition over the 50-year-long follow-up period [21, 31] providing a context for sample size considerations. Even though some associations were found in whole-day analyses, a low number of cases might have led to the observed non-significant associations, especially in the analyses of occupational domain. This outcome is unfortunate, since it would have been interesting to determine whether the SB of girls with higher behavioral activity accumulated, especially at work. In this study, conclusions about causal relationships cannot be drawn due to the observational characteristics of the follow-up study. Despite all limitations, the value of this study lies in the uniquely long follow-up period of five decades that provides new insights to the current literature on the links between personality characteristics and PA and SB.

At the conceptual level, although socioemotional behavior and temperament display some similarities, they are two distinct concepts, where the former shifts attention to socialization experiences and is more situation-specific [21]. However, socioemotional behavior was used as a conceptual counterpart of child temperament in this study because the children’s temperament measures developed by Rothbart et al. [19] were not available at the time of the first data collection in 1968 [21].

Despite the multiple strengths of the accelerometer measurement, the device is not optimal for estimating non-step-based activities, such as cycling and gym training [34]. Additionally, the intensity cut-points are based on absolute intensity, which does not consider individual experiences of physical load even though they are highly correlated with VO_2max_ [35]. Information on body posture was not analyzed either. Thus, SB in the current study may involve some activities done in a standing position, though the consensus is that SB refers only to the time spent sitting, reclining, or lying [8].

It should also be noted that accelerometer data were collected between March 2020 and May 2021, that is, amid the COVID-19 pandemic. During the declared state of emergency in Finland (March 16, 2020–June 16, 2020), gatherings of more than 10 people were restricted, public indoor sports facilities (e.g., swimming halls) were closed, and remote work was strongly recommended, among other measures [50, 51]. However, data collection was suspended during that period and continued from June 2, 2020 onwards because of the favorable situation for proceeding with the measurements. Sensitivity analyses on the current sample also revealed that exclusion of those participants whose measurement overlapped with the state of emergency at any point (N = 11) did not change the results.

Although the pandemic continued to affect daily life after the state of emergency, the effect on accelerometer-based PA in the present study is likely to be trivial. First, compared internationally, the pandemic situation in Finland during the data collection period was mild in terms of the incidence of COVID-19 and the restrictions [33]. For example, a curfew was never imposed by Finnish authorities, and thus, restricted indoor activities were replaced by outdoor activities, especially walking, in adults [50]. Second, international comparisons during the pandemic have suggested that even a partial lockdown did not affect device-based PA and that the effect of restriction orders on PA diminished after a couple of weeks [52]. Compared to self-reports, accelerometers capturing habitual and incidental movements during the day [47] are also expected to be less prone to possible changes in deliberate exercise. This standpoint is supported by the relatively similar MVPA and SB levels of the study sample compared to those reported among the Finnish population aged 50–69 years before the pandemic [53]. Third, although the pandemic-caused pressure related to housework and caregiving on women has been discussed, it is not likely to affect the current sample. In Finland, women living in the transition stage to late adulthood rarely have childrearing duties and their employment rate is equal to that of men [54].

## Conclusions

This study extends the previous literature by suggesting that child socioemotional behavior and adult temperament have predictive value for accelerometer-measured PA and SB in women after decades. The behavioral activity of girls predicted higher levels of daily SB in late adulthood, but the association was attenuated when their own occupational status was taken into account. The negative affectivity of women predicted lower daily and leisure-time MVPA in late adulthood. Although few weak associations of socioemotional behavior and temperament with PA and SB were detected in women, they were observed over several decades, and thus, deserve attention in future studies. In light of these findings, health professionals may also be sensitive to individual characteristics, such as a tendency to experience more negative emotions, when doing health counseling or planning for health-promoting interventions targeting PA and SB. Future research should also address ways of promoting especially leisure-time PA among those high in negative affectivity.

The generalization of the results obtained from a native Finnish sample born in 1959 to other populations, age groups and later-born cohorts should be done with caution. Moreover, future studies should, in general, involve larger and more diverse samples as well as utilize domain-specific approaches and powerful longitudinal designs to investigate causal relationships. Particularly based on this study, it would be a major interest to shed light on the possible mechanisms, such as career-related variables, between child characteristics and later PA and SB. In addition, this research topic has traditionally been examined based on single dimensions. However, since a person can score high or low in several socioemotional or temperament dimensions at the same time, analyzing the combinations of these dimensions would be a more comprehensive approach, consequently gaining a deeper understanding of the complex associations between the variables of interest.

## Electronic supplementary material

Below is the link to the electronic supplementary material.

Supplementary Material 1

Supplementary Material 2

## Data Availability

Owing to the sensitivity of the data and privacy of the participants, the law dictates that the data cannot be openly shared. Except for the most recent, age 61 data, the data are stored in the Finnish Social Science Data Archive (FSD) (https://www.fsd.uta.fi/en/). The data analyses that support the findings of the present article are available from the corresponding author upon reasonable request. Pseudonymized datasets are available to external collaborators upon agreement on the terms of data use and publication of results. To request the data please contact the Principal Investigator Dr. Katja Kokko (katja.r.kokko@jyu.fi).

## List of Abbreviations

PA: Physical activity
MVPA: Moderate-to-vigorous physical activity
SB: Sedentary behavior
