# Supplementary material for: Child socioemotional behavior and adult temperament as predictors of physical activity and sedentary behavior in late adulthood
Source: BMC Public Health. 2023 Jun 19;23:1179. doi: 10.1186/s12889-023-16110-y (PMC10280976; doi:10.1186/s12889-023-16110-y)
Supplement: Supplementary file 1 — Supplementary Material 1 [file 12889_2023_16110_MOESM1_ESM.docx]

# **Supplementary information**

Additional file 1: **Table S1.** Correlation matrix showing Pearson Bivariate correlations: Correlations for women (N = 58–78) above the diagonal and correlations for men (N = 35–58) below the diagonal. **Table S2.** Linear regressions of child socioemotional dimensions predicting leisure-time MVPA and SB. **Table S3.** Linear regressions of child socioemotional dimensions predicting occupational MVPA and SB. **Table S4.** Linear regressions of adult temperament dimensions predicting leisure-time MVPA and SB. **Table S5.** Linear regressions of adult temperament dimensions predicting occupational MVPA and SB.
