# Supplementary material for: Child socioemotional behavior and adult temperament as predictors of physical activity and sedentary behavior in late adulthood
Source: BMC Public Health. 2023 Jun 19;23:1179. doi: 10.1186/s12889-023-16110-y (PMC10280976; doi:10.1186/s12889-023-16110-y)
Supplement: Supplementary file 2 — Supplementary Material 2 [file 12889_2023_16110_MOESM2_ESM.docx]

# **Table S1.** Correlation matrix showing Pearson Bivariate correlations: Correlations for women (N = 58–78) above the diagonal and correlations for men (N = 35–58) below the diagonal.

| Variable | 1 | 2 | 3 | 4 | 5 | 6 | 7 | 8 | 9 | 10 | 11 | 12 | 13 | 14 | 15 | 16 | 17 | 18 | 19 |
| --- | --- | --- | --- | --- | --- | --- | --- | --- | --- | --- | --- | --- | --- | --- | --- | --- | --- | --- | --- |
| 1. Whole-day MVPA 61 | 1 | .91** | .64** | -.37** | -.23* | -.15 | .05 | .10 | -.02 | .21 | .10 | -.28* | .11 | .34** | .02 | .09 | .30** | .27* | .08 |
| 2. Leisure-time MVPA 61 | .91** | 1 | .33* | -.24* | -.05 | -.04 | .04 | .11 | -.05 | .28* | .10 | -.27* | .17 | .30** | .23*^a^ | .01 | .22 | .24* | .11 |
| 3. Occupational MVPA 61 | .66** | .28 | 1 | -.50** | -.12 | -.31* | .02 | -.03 | .04 | .05 | .02^a^ | -.18^b^ | -.02 | .17 | .03 | .22 | .12 | .19 | -.14 |
| 4. Whole-day SB 61 | -.42** | -.36** | -.50** | 1 | .46** | .59** | .18 | .07 | -.02 | -.03 | .13 | .06 | -.02 | .30** | .19 | -.03 | .04 | -.17 | .30** |
| 5. Leisure-time SB 61 | -.18 | .03 | -.18 | .50** | 1 | -.34** | .09 | -.03 | -.02 | .06 | -.01 | .09 | .02 | -.04 | .87**^a^ | -.62** | -.34** | -.16^a^ | .05 |
| 6. Occupational SB 61 | -.35* | -.21 | -.42** | .71** | -.05 | 1 | .04 | .09 | -.01 | .08 | .08 | -.07^a^ | .01 | .21 | -.35** | .66** | .23 | .02 | .18 |
| 7. Behavioral activity 8 | .00 | .17 | -.20 | .03 | .16 | .24 | 1 | -.04 | -.17 | -.03^b^ | .01 | -.02 | -.12^b^ | -.02 | .00^a^ | -.09 | .07 | .24* | .26* |
| 8. Well-controlled behavior 8 | -.09 | -.05 | -.22 | -.12 | -.25 | .22 | .12 | 1 | -.29*^a^ | -.15 | -.04 | .07 | -.17 | .06 | -.04 | .09 | -.13 | -.10 | -.08 |
| 9. Negative emotionality 8 | -.03 | -.03 | .00 | -.01 | .13 | -.08 | -.26* | -.52**^a^ | 1 | -.14 | .06 | .04 | .06 | .10 | .03 | -.04 | -.04 | -.18 | -.06 |
| 10. Surgency 42 | .05 | .14 | -.18 | .04 | .09 | .19 | .41**^b^ | -.08 | -.06 | 1 | .23 | -.32** | .45*^a^ | .00 | .12 | .05 | .09 | .13 | .30* |
| 11. Effortful control 42 | -.04 | .02 | -.30^a^ | .05 | -.22 | .38* | -.09 | .14 | -.15 | .09 | 1 | -.34*^c^ | .06 | .05 | -.01 | -.08^a^ | -.01 | .12 | .12 |
| 12. Negative affectivity 42 | -.02 | -.13 | .32^b^ | -.05 | .07 | -.42*^a^ | .01 | -.02 | .05 | -.29* | -.73**^c^ | 1 | .10 | -.05 | -.03 | .00 | -.03 | -.15 | -.13 |
| 13. Orienting sensitivity 42 | .08 | .11 | -.05 | .02 | .08 | -.02 | .32*^b^ | -.15 | -.12 | .68**^a^ | .06 | -.15 | 1 | -.14 | .01 | -.03 | .08a | .09 | .08 |
| 14. Whole-day wear time 61 | .30* | .17 | -.01 | .35** | -.16 | .34* | -.13 | .05 | -.09 | -.08 | .01 | .04 | -.03 | 1 | .13^a^ | .12 | .38**^a^ | .06 | .13^b^ |
| 15. Leisure-time wear time 61 | .23 | .49**^a^ | .01 | .02 | .77**^a^ | -.22 | .27*^a^ | -.16 | .17 | .14 | -.21 | .01 | .11 | -.17^a^ | 1 | -.64** | -.27* | -.08 | .02 |
| 16. Occupational wear time 61 | -.09 | -.14 | .03 | -.06 | -.61** | .48** | .13 | .36* | -.16 | .06 | .31^a^ | -.27 | -.10 | .31 | -.67** | 1 | .20 | .09 | -.17 |
| 17. Self-rated health 61 | .30* | .28* | .05 | -.14 | -.22 | .03 | -.04 | .10 | -.18 | .25 | .15 | -.20 | .34**^a^ | .09^a^ | -.13 | .21 | 1 | .26* | .32** |
| 18. Parents’ occupational status 8 | .14 | .24 | -.05 | .00 | .15^a^ | .04 | .15 | .15 | -.29* | .01 | .09 | -.03 | -.07 | -.02 | .16 | .15 | .10 | 1 | .18 |
| 19. Participant’s occupational status 61 | .25* | .32* | -.12 | -.13 | -.08 | .37* | .18 | .08 | -.02 | .25 | .21 | -.12 | .06 | -.23^b^ | .12 | .09 | .17 | .30* | 1 |
| *Note*. MVPA = moderate-to-vigorous physical activity, SB = sedentary behavior.  * p < .05, ** p < .01  Fisher’s z-test: ^a^ = p < .05, ^b^ = p < .01, ^c^ = < .001 | | | | | | | | | | | | | | | | | | | |

# **Table S2.** Linear regressions of child socioemotional dimensions predicting leisure-time MVPA and SB.

|  | | Leisure-time MVPA | | | | | | | | | Leisure-time SB | | | | | | | | |  |
| --- | --- | --- | --- | --- | --- | --- | --- | --- | --- | --- | --- | --- | --- | --- | --- | --- | --- | --- | --- | --- |
|  | | Model 1 ^a^ | | | Model 2 ^b^ | | | Model 3 ^c^ | | | Model 1 ^a^ | | | Model 2 ^b^ | | | Model 3 ^c^ | | |  |
|  | | β | p | R^2^_adj._ | β | p | R^2^_adj._ | β | p | R^2^_adj._ | β | p | R^2^_adj._ | β | p | R^2^_adj._ | β | p | R^2^_adj._ |  |
| Women | |  |  | .04 |  |  | .16 |  |  | .14 |  |  | .75 |  |  | .77 |  |  | .76 |  |
|  | Behavioral activity | .04 | .701 |  | –.01 | .911 |  | –.01 | .906 |  | .08 | .158 |  | .11 | .071 |  | .09 | .130 |  |  |
|  | Well-controlled behavior | .07 | .537 |  | .17 | .140 |  | .16 | .160 |  | –.01 | .923 |  | –.03 | .606 |  | –.03 | .659 |  |  |
|  | Negative emotionality | –.03 | .799 |  | .02 | .827 |  | .02 | .883 |  | –.03 | .570 |  | –.06 | .300 |  | –.06 | .357 |  |  |
| Men | |  |  | .25 |  |  | .37 |  |  | .41 |  |  | .60 |  |  | .62 |  |  | .63 |  |
|  | Behavioral activity | –.02 | .867 |  | .00 | .976 |  | –.08 | .477 |  | –.05 | .547 |  | –.07 | .438 |  | –.03 | .766 |  |  |
|  | Well-controlled behavior | –.06 | .619 |  | –.11 | .364 |  | –.08 | .486 |  | –.17 | .078 |  | –.13 | .178 |  | –.13 | .190 |  |  |
|  | Negative emotionality | –.16 | .248 |  | –.09 | .507 |  | –.12 | .350 |  | –.10 | .289 |  | –.10 | .318 |  | –.08 | .440 |  |  |
| *Note*. Socioemotional dimensions analyzed in same regression models. Women N = 78, men N = 63. Square root transformation of leisure-time MVPA was used. MVPA = moderate-to-vigorous physical activity, SB = sedentary behavior, β = standardized beta-coefficients, p = p-value, R^2^_adj._ = adjusted coefficient of determination for the model. Statistically significant results bolded. None of the standardized beta-coefficients were statistically significant after the Benjamini–Hochberg correction. | | | | | | | | | | | | | | | | | | | |  |
|  |  |  |  |  |  |  |  |  |  |  |  |  |  |  |  |  |  |  |  |  |
| ^a^ Model 1: Adjusted for accelerometer wear time | | | | | | | | | | | | | | | | | | | |  |
| ^b^ Model 2: Adjusted for accelerometer wear time, season, parents’ occupational status, and self-rated health | | | | | | | | | | | | | | | | | | | |  |
| ^c^ Model 3: Adjusted for accelerometer wear time, season, parents’ occupational status, self-rated health, and participant’s occupational status | | | | | | | | | | | | | | | | | | | |  |

# **Table S3.** Linear regressions of child socioemotional dimensions predicting occupational MVPA and SB.

|  | | Occupational MVPA | | | | | | | | | Occupational SB | | | | | | | | |  |
| --- | --- | --- | --- | --- | --- | --- | --- | --- | --- | --- | --- | --- | --- | --- | --- | --- | --- | --- | --- | --- |
|  | | Model 1 ^a^ | | | Model 2 ^b^ | | | Model 3 ^c^ | | | Model 1 ^a^ | | | Model 2 ^b^ | | | Model 3 ^c^ | | |  |
|  | | β | p | R^2^_adj._ | β | p | R^2^_adj._ | β | p | R^2^_adj._ | β | p | R^2^_adj._ | β | p | R^2^_adj._ | β | p | R^2^_adj._ |  |
| Women | |  |  | .09 |  |  | .19 |  |  | .18 |  |  | .40 |  |  | .45 |  |  | .55 |  |
|  | Behavioral activity | .08 | .535 |  | .03 | .802 |  | .05 | .726 |  | .12 | .260 |  | .13 | .191 |  | .05 | .593 |  |  |
|  | Well-controlled behavior | .02 | .900 |  | –.06 | .643 |  | –.06 | .637 |  | .05 | .621 |  | .18 | .123 |  | .17 | .103 |  |  |
|  | Negative emotionality | .06 | .628 |  | .08 | .554 |  | .05 | .694 |  | .06 | .583 |  | .07 | .523 |  | .03 | .778 |  |  |
| Men | |  |  | –.04 |  |  | –.15 |  |  | –.19 |  |  | .17 |  |  | .07 |  |  | .15 |  |
|  | Behavioral activity | –.03 | .850 |  | –.02 | .905 |  | –.02 | .911 |  | .18 | .244 |  | .17 | .322 |  | .02 | .922 |  |  |
|  | Well-controlled behavior | –.27 | .175 |  | –.32 | .146 |  | –.26 | .256 |  | .04 | .808 |  | .06 | .751 |  | .08 | .675 |  |  |
|  | Negative emotionality | –.21 | .255 |  | –.22 | .284 |  | –.19 | .398 |  | .04 | .823 |  | .02 | .918 |  | –.06 | .740 |  |  |
| *Note*. Socioemotional dimensions analyzed in same regression models. Women N = 61, men N = 38. Cube root transformation of occupational MVPA was used. MVPA = moderate-to-vigorous physical activity, SB = sedentary behavior, β = standardized beta-coefficients, p = p-value, R^2^_adj._ = adjusted coefficient of determination for the model. Statistically significant results bolded. None of the standardized beta-coefficients were statistically significant after the Benjamini–Hochberg correction. | | | | | | | | | | | | | | | | | | | |  |
|  |  |  |  |  |  |  |  |  |  |  |  |  |  |  |  |  |  |  |  |  |
| ^a^ Model 1: Adjusted for accelerometer wear time | | | | | | | | | | | | | | | | | | | |  |
| ^b^ Model 2: Adjusted for accelerometer wear time, season, parents’ occupational status, and self-rated health | | | | | | | | | | | | | | | | | | | |  |
| ^c^ Model 3: Adjusted for accelerometer wear time, season, parents’ occupational status, self-rated health, and participant’s occupational status | | | | | | | | | | | | | | | | | | | |  |

# **Table S4.** Linear regressions of adult temperament dimensions predicting leisure-time MVPA and SB.

|  | | Leisure-time MVPA | | | | | | Leisure-time SB | | | | | |  |
| --- | --- | --- | --- | --- | --- | --- | --- | --- | --- | --- | --- | --- | --- | --- |
|  | | Model 1 ^a^ | | | Model 2 ^b^ | | | Model 1 ^a^ | | | Model 2 ^b^ | | |  |
|  | | β | p | R^2^_adj._ | β | p | R^2^_adj._ | β | p | R^2^_adj._ | β | p | R^2^_adj._ |  |
| Women | |  |  | .12 |  |  | .19 |  |  | .74 |  |  | .75 |  |
|  | Surgency | .13 | .337 |  | .14 | .321 |  | –.01 | .904 |  | .00 | .986 |  |  |
|  | Effortful control | –.01 | .905 |  | –.03 | .774 |  | .04 | .559 |  | .02 | .770 |  |  |
|  | Negative affectivity | **–.27** | **.040** |  | **–.25** | **.045** |  | .12 | .082 |  | .11 | .117 |  |  |
|  | Orienting sensitivity | .09 | .509 |  | .04 | .760 |  | .00 | .983 |  | –.01 | .848 |  |  |
| Men | |  |  | .27 |  |  | .47 |  |  | .56 |  |  | .62 |  |
|  | Surgency | .04 | .821 |  | .02 | .887 |  | –.02 | .880 |  | .00 | .998 |  |  |
|  | Effortful control | .14 | .432 |  | .18 | .288 |  | –.04 | .771 |  | –.08 | .587 |  |  |
|  | Negative affectivity | .03 | .872 |  | .12 | .489 |  | .02 | .863 |  | –.04 | .775 |  |  |
|  | Orienting sensitivity | .04 | .777 |  | –.12 | .410 |  | .01 | .934 |  | .07 | .575 |  |  |
| *Note*. Temperament dimensions analyzed in same regression models. Women N = 73, men N = 57. Square root transformation of leisure time MVPA was used. MVPA = moderate-to-vigorous physical activity, SB = sedentary behavior, β = standardized beta-coefficients, p = p-value, R^2^_adj._ = adjusted coefficient of determination for the model. Statistically significant results bolded. None of the standardized beta-coefficients were statistically significant after the Benjamini–Hochberg correction. | | | | | | | | | | | | | |  |
|  |  |  |  |  |  |  |  |  |  |  |  |  |  |  |
| ^a^ Model 1: Adjusted for accelerometer wear time | | | | | | | | | | | | | |  |
| ^b^ Model 2: Adjusted for accelerometer wear time, season, participant’s occupational status, and self-rated health | | | | | | | | | | | | | |  |

# **Table S5.** Linear regressions of adult temperament dimensions predicting occupational MVPA and SB.

|  | | Occupational MVPA | | | | | | Occupational SB | | | | | |  |
| --- | --- | --- | --- | --- | --- | --- | --- | --- | --- | --- | --- | --- | --- | --- |
|  | | Model 1 ^a^ | | | Model 2 ^b^ | | | Model 1 ^a^ | | | Model 2 ^b^ | | |  |
|  | | β | p | R^2^_adj._ | β | p | R^2^_adj._ | β | p | R^2^_adj._ | β | p | R^2^_adj._ |  |
| Women | |  |  | .11 |  |  | .11 |  |  | .40 |  |  | .50 |  |
|  | Surgency | .14 | .342 |  | .21 | .176 |  | –.01 | .957 |  | –.12 | .321 |  |  |
|  | Effortful control | –.08 | .559 |  | –.11 | .436 |  | .13 | .270 |  | .08 | .444 |  |  |
|  | Negative affectivity | –.12 | .409 |  | –.07 | .649 |  | –.03 | .787 |  | –.05 | .644 |  |  |
|  | Orienting sensitivity | .03 | .849 |  | –.02 | .897 |  | .03 | .765 |  | .06 | .600 |  |  |
| Men | |  |  | .02 |  |  | -.05 |  |  | .31 |  |  | .55 |  |
|  | Surgency | –.25 | .338 |  | –.16 | .570 |  | .22 | .316 |  | .06 | .734 |  |  |
|  | Effortful control | –.20 | .514 |  | –.10 | .778 |  | .00 | .995 |  | .12 | .628 |  |  |
|  | Negative affectivity | .17 | .596 |  | .26 | .468 |  | –.27 | .310 |  | –.16 | .508 |  |  |
|  | Orienting sensitivity | .18 | .472 |  | .00 | .991 |  | –.18 | .388 |  | .15 | .443 |  |  |
| *Note*. Temperament dimensions analyzed in same regression models. Women N = 58, men N = 35. Cube root transformation of occupational MVPA was used. MVPA = moderate-to-vigorous physical activity, SB = sedentary behavior, β = standardized beta-coefficients, p = p-value, R^2^_adj._ = adjusted coefficient of determination for the model. Statistically significant results bolded. None of the standardized beta-coefficients were statistically significant after the Benjamini–Hochberg correction. | | | | | | | | | | | | | |  |
|  |  |  |  |  |  |  |  |  |  |  |  |  |  |  |
| ^a^ Model 1: Adjusted for accelerometer wear time | | | | | | | | | | | | | |  |
| ^b^ Model 2: Adjusted for accelerometer wear time, season, participant’s occupational status, and self-rated health | | | | | | | | | | | | | |  |
